# Who stays? Who goes? Motivation and tendency to drop out in music schools

**DOI:** 10.3389/fpsyg.2024.1378843

**Published:** 2024-08-07

**Authors:** Martin Wieser, Verena Novak-Geiger, Florian H. Müller

**Affiliations:** ^1^School of Education, University of Klagenfurt, Klagenfurt, Austria; ^2^Institute of Instructional and School Development, University of Klagenfurt, Klagenfurt, Austria

**Keywords:** instrumental music instruction, self-determination theory, basic psychological needs, tendency to drop-out, parental involvement

## Abstract

Based on self-determination theory, this study examined the extent to which the satisfaction of the basic psychological needs for autonomy, competence, and social relatedness in instrumental lessons explain the quality and quantity of motivation, which are responsible for persistence and dropout in music schools. This study also investigated whether parental involvement contributes to dropout. A total of 140 music students from Austria (37.16% male, 62.1% female, 0.8% diverse) were surveyed using a quantitative questionnaire. The central variables are the tendency to dropout (dependent variable) and, as predictors, the motivational regulation styles, the satisfaction of basic psychological needs in the classroom and parental involvement. The results of a structural equation model indicated that satisfaction of basic needs in class and parental involvement, mediated by motivation, predicted dropout tendencies. Autonomous motivation in lessons is negatively associated and controlled motivation is positively associated with the tendency to drop out of music schools. Satisfaction of basic psychological needs during lessons and parental involvement predicts autonomous motivation. However, basic psychological needs cannot predict controlled motivation but parental involvement can predict controlled motivation to a limited extent. Finally, this study emphasizes the practical importance of need satisfaction and parental involvement in motivation and continuing to play a musical instrument.

## 1 Introduction

Learning to play musical instruments is a rewarding experience for both children and teenagers and requires practice and hard work to achieve proficiency. However, not all individuals find it equally enjoyable and may struggle to maintain motivation; over half of music students stop playing their instruments by the age of 17 years (Ruth and Müllensiefen, 2021). The reasons for the individual differences in motivation may lie in the quality of music instruction or support provided by the social environment (McPherson et al., 2012). Therefore, drawing on self-determination theory (SDT; Ryan, 2023), this study examined the reasons for the tendency to dropout of music schools. According to SDT, basic psychological need satisfaction (BPNS) is fundamental to the quality of motivation that can predict persistence or dropout (Evans et al., 2013; Ricard and Pelletier, 2016).

Over the last two decades, an increasing number of studies have emerged on motivational conditions, processes, and outcomes in the field of music (for a summary, see Evans, 2015). However, studies that explicitly concern the motivational aspects of learning a musical instrument (e.g., Küpers et al., 2014; Evans, 2015) are scarce, particularly when exploring persistence and dropout from music schools (Ruth and Müllensiefen, 2021). Nevertheless, previous studies have indicated that parental involvement, similar to the formal education system, is important in motivating children to play musical instruments (McPherson et al., 2012; Evans, 2015). The present study explored whether BPNS in instrumental music lessons in music schools and music-related parental involvement could explain motivational regulation and, ultimately, music school dropout tendency.

Music schools in Austria, where this study was conducted, are not integrated into the formal education system and are financed by public funds (approximately 3/4) and school fees (approximately 1/4). There are a total of about 380 music schools throughout Austria and music school teachers are employed by the federal state and have completed a university or conservatory degree in instrumental music or vocal music education. Music school lessons are an extracurricular activity that typically occurs during the afternoon. Children, adolescents, and adults learn to play musical instruments at music schools that offer major subjects (instrumental instruction and vocal instruction) and minor subjects (music theory, ensemble playing and orchestral playing). A total of 205,000 individuals took lessons in Austrian music schools during 2020/2021 (KOMU, 2024). Usually, lessons in music schools occur weekly in one-to-one and group lessons. Outside of music schools, it is possible to receive private instrumental or singing lessons. However, these are not within the scope of music schools and were not included in the present study.

## 2 Self-determination theory

**Figure 1:**
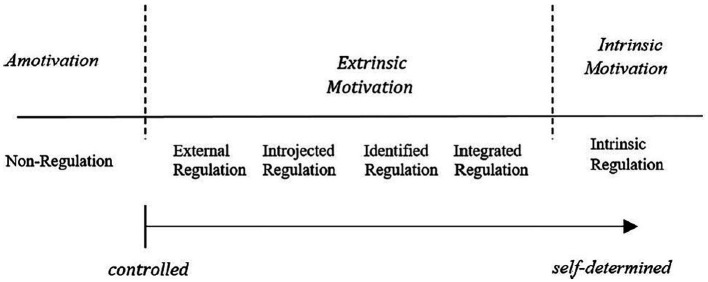
Continuum of self-determination (based on Deci and Ryan, 2002, p. 16).

Compared with other motivation theories, such as trait theory approaches or concepts that can be assigned to the tradition of cognitive action theories, SDT is based on the assumption of dynamic personality concepts (cf. Krapp et al., 2014). SDT (Ryan and Deci, 2017; Ryan, 2023) is a functional theory of motivation that allows analyzing motivationally relevant personal and environmental conditions, motivational processes, and outcomes in context, particularly in the field of education. It proposes different types of motivation along a continuum, as displayed in Figure 1: amotivation, external regulation, introjected regulation, identified regulation, integrated regulation, and intrinsic motivation. External regulation aligns with the traditional concept of extrinsic motivation and involves pursuing rewards or avoiding unfavorable outcomes. For a music student, external regulation is exhibited when students prepare well for a lesson because they are afraid that the teacher might give them negative feedback. However, this regulation lacks self-determination. Introjected regulation is another type of extrinsically motivated behavior where the individual has taken in external factors from others that the person is not entirely consensual with. For example, individuals with introjected motivation are motivated by feelings of guilt, fear of disapproval, and seeking ego improvement. In an educational context, when students learn because they are afraid of embarrassment in the classroom, experience guilt toward their parents, or want to increase their self-esteem by outperforming others, this is known as introjected regulation. In SDT, a distinction has been made between positive and negative introjected regulation, and between its approach and avoidance aspects (Pelletier et al., 2013; Gagné et al., 2015). The approach aspect relates to increasing self-esteem (e.g., being better than others), whereas the avoidance aspect relates to avoiding guilt or acting out of shame. Identified regulation is a more self-determined form of extrinsic motivation wherein the value of external behavior is accepted or perceived as important. For example, a music student identifies with the self-imposed goal of wanting to play in a band and, therefore, practices regularly. The action is still extrinsically motivated; however, it is characterized by significantly more autonomy than the other two regulatory styles of extrinsic motivation. Finally, integrated motivation is the most autonomous form of extrinsic motivation because identified regulations are completely internalized and integrated within the self after evaluation and congruency with the individual’s other needs and values. At the end of the continuum of self-determination is intrinsic motivation, which can be described as the prototype of self-determination and is associated with pleasure, interest and a desire to learn.

Additionally, three basic psychological needs (BPN)—autonomy, competence, and social relatedness—are central to motivated behavior (cf. Vandenkerckhove et al., 2019; Ryan, 2023) because establishing intrinsic motivation requires the fulfillment of the following needs. First, autonomy refers to the need to regulate one’s actions and experiences. A characteristic feature of autonomy is that actions are promoted by oneself and are consistent with one’s interests and values. For example, when music students have a say in the selection of pieces in instrumental music lessons. Second, competence refers to the need to experience accomplishment and mastery in central life contexts. The need for competence is closely associated with the experience of self-efficacy (Bandura, 1971) and is expressed as the feeling of being able to do something or becoming better. In educational institutions, structures that promote competence-oriented feedback and transparent requirements are particularly conducive to the development of competences. Third, social relatedness refers to the need for belonging and feeling significant as well as contributing to a social group. Feeling connected with and contributing to others is essential (Baumeister and Leary, 1995). This can be manifested in music lessons through the teacher being perceived as a caring and secure reference. Individuals with BPNS are creative, productive, and compassionate. When fulfilled, students feel in control of their own lives, skilled in completing tasks ahead, and part of a concerned environment; hence, motivational behavior develops (Vansteenkiste et al., 2020).

## 3 Current state of research

### 3.1 SDT and instrumental music

A large number of studies have attested to the relevance of SDT in education. For example, numerous empirically validated effects have been identified concerning the affect, cognition, and behavior of autonomously motivated actions and BPNS (Taylor et al., 2014; Núñez and León, 2016; Ryan, 2023). Compared with other areas of education, motivation research has only recently focused on music education and has increasingly examined the conditions, processes, and outcomes of self-determined motivation in music education (MacIntyre et al., 2018; Miksza et al., 2019; Kingsford-Smith and Evans, 2021). Additionally, motivational studies concerning instrumental teaching have been increasingly presented (Comeau et al., 2015; Evans, 2015; Schatt, 2018; Evans and Liu, 2019; Wieser and Müller, 2022; Kavčič Pucihar et al., 2024). According to Evans (2015), the SDT framework is suitable for examining conditions, outcomes, and motivation in music education. Perceived satisfaction with autonomy, competence, and social relatedness is crucial for motivation prediction. Renwick and McPherson (2002) demonstrated the importance of autonomy in the quality of learning and performance in music education. Furthermore, belief in one’s competence plays a crucial role in continuing to learn and play a musical instrument, as most individuals engage in music-making as a voluntary activity and often stop playing if they do not perceive sufficient competence (O’Neill and Sloboda, 1997). Social relatedness is associated with self-determined forms of motivation to play instruments (Evans et al., 2013; Wieser, 2018; Wieser and Müller, 2019). Additionally, children whose basic psychological needs are not met or are inadequately met are more likely to give up playing an instrument (Evans et al., 2013; Evans, 2015; Kavčič Pucihar et al., 2024). The central importance of BPNS in motivation was recently demonstrated in a study concerning online music lessons (Kruse et al., 2013). The significance of autonomy and social integration is particularly evident in the online context. Evans and Liu (2019) demonstrated that BPNS is essential for autonomous forms of motivation when playing instruments in a high school orchestra. Evans (2015) observed that social relatedness continues to play a central role in the development of competence in later life. Another study that distinguished between different musical genres indicated that popular jazz and folk musicians experienced more intrinsic motivation and autonomy than those dedicated to classical music (de Bézenac and Swindells, 2009). Classical music culture tends to be more controlled than other types of music cultures. In summary, BPNS is not only important for the quality of motivation in music lessons and when learning an instrument but also for relevant outcomes such as resistance, identification, and sustainable learning. Therefore, SDT is particularly suitable for studies on dropout behavior.

### 3.2 Parents and their influence on music

Families play an essential role in children’s and adolescents’ motivational development in instrumental music. Music-related interactions within the family, particularly with parents, directly influence the motivation to learn to play musical instruments. Involvement has proven to be a particularly compelling explanation for this phenomenon. In the field of music, this could refer to parents practicing with their children, taking part in music lessons, and attending concerts (McPherson, 2009). Additionally, parental involvement leads to higher achievement and better motivational development (cf. Davidson et al., 1996; Zdzinski, 1996), particularly when this involvement encourages autonomy and less control (McPherson and Davidson, 2002; Kavčič Pucihar et al., 2024). Furthermore, a connection between musical involvement and self-concept has been discovered (Simpkins et al., 2010). The importance of parents’ place in music is considered an important factor in the genesis of musical motivation as a whole and in playing instruments (Leung and McPherson, 2010; McPherson and Hendricks, 2010; McPherson and O’Neill, 2010; McPherson et al., 2012). Parents’ expectations and beliefs concerning their children’s competencies and parental feedback are closely associated with higher achievement and motivation (McPherson and Davidson, 2002; McPherson, 2009). Parents encourage and strengthen their children’s self-efficacy through feedback (Schunk and Meece, 2006). Previous studies (e.g., Cheng and Southcott, 2023) have shown that students’ engagement in practice is influenced by parental support. Some individuals viewed their parents in a monitoring role, whereas others found parental feedback helpful. According to Sichivitsa (2007), children tend to develop a positive self-concept based on their parents’ beliefs and conceptions. Holster (2023) found a strong positive relationship between parents’ influence, needs satisfaction, and task value in the music learning domains.

### 3.3 Dropout and music education

According to early studies on dropout from music schools in Austria (Sonderegger, 1996) and Germany (Switlick and Bullerjahn, 1999), the main reasons for dropping out were lack of motivation and loss of motivation, competing leisure activities and the associated lack of time, as well as criticism of music teachers’ behavior. Recently, a growing body of research examined the relationship between basic psychological needs according to self-determination theory (SDT) and music dropout. Previous studies have examined ceasing music (activities) in adults through retrospective interviews and have highlighted numerous obstacles hindering lifelong engagement in music (Pitts and Robinson, 2016). In a recent qualitative interview study, Kavčič Pucihar et al. (2024) demonstrated that, in addition to BPNS, the presence of competing extracurricular interests or a lack of interest in music theory and solfège can also explain dropout. Furthermore, a quantitative survey involving children, conducted during their participation in a school music program and after discontinuation, indicated that BPNS tended to be notably low just before cessation compared with earlier assessment points (Evans et al., 2013). Additionally, StGeorge’s (2004) comprehensive review identified several discontinuations of musical education contributing factors. These encompass sociological and sociopsychological elements, such as the family’s social and economic status, perceived support, practice time, personal beliefs concerning competence, and external motivation, all of which play a role in the decision to discontinue music education. While extant studies often centered on music as a school subject, particularly instrumental music, only a limited number of studies have explored the intersection of SDT and dropout/ceasing musical activities, as exemplified by Evans et al. (2013). Notably, empirical studies examining motivation in extracurricular (instrumental) music setting contexts are scarce. One area of interest involves the examination of the influence of parents and families on music and musical activities. However, a notable absence of empirical studies grounded in SDT remains within this domain. This study aimed to investigate extracurricular instrumental music learning in Austrian music schools, focusing on basic psychological need fulfillment and parental involvement as explanatory factors for the continuation or tendency to dropout in music schools. Based on the current state of research, the following hypotheses were derived:

1. The higher the BPNS, the more pronounced the autonomous forms of motivation. Moderate associations are expected in this study. BPNS exhibits a weak or nonexistent correlation with controlled forms of motivation.
2. The higher the students’ perceptions of parental involvement, the higher their autonomous forms of motivation will be. Moderate correlations are to be expected here. Parental involvement tends to negatively correlate with controlled regulatory styles of motivation.
3. The higher the students’ autonomous forms of motivation, the lower their tendency to leave music school. Moderate correlations are expected. According to this hypothesis, controlled forms of motivation can also explain the tendency to dropout.
4. Parental involvement and BPNS can explain the tendency to dropout mediated by motivational regulation. Thus, a structural equation model was used to examine the extent to which parental involvement and BPNS could explain the tendency to drop out of music schools. Direct explanatory paths from parental involvement or BPNS to the tendency to drop out are expected to be small or zero.

## 4 Materials and methods

### 4.1 Participants

The sample comprised 140 music students (37.1% male, 62.1% female, 0.8% diverse) aged 8–27 years (*M*_age_ = 12.86, SD = 3.89) and was conducted at three music schools in Austria. The most prominent and represented instrument groups were woodwinds (26.2%), followed by keyboard instruments (20.6%), and strings (18.4%). On average, the musical students in this study had four musical instruments at home. Of the participants, 80.9% had at least one family member who played an instrument, and 76.9% played with family members at least once per week. Furthermore, 93.0% had friends who played an instrument. Additionally, 24.8% practiced 0–15 min per day, 48.2% practiced 15–30 min, 19.9% 30–45 min, and 5.0% practiced more than 45 min per day. The sample size was estimated using G*Power software (Version 3.1.9.2; Faul et al., 2009), with ƒ = 0.15 (small effect size), β = 0.95, and α = 0.05. The recommended sample size was 138; therefore, this study’s sample size of 140 participants was adequate.

### 4.2 Procedure

This study was approved by the institutional ethics committee (University of Klagenfurt, No. 2022–068). The participants were recruited via the music school’s principal. Therefore, the research team handed out the questionnaires to the school principal with a request to forward the forms to the teachers, who handed them out to their students during a regular music lesson. The participants were informed that the questionnaires were anonymous and that their data would not be available to any teacher, school principal, or third party. Furthermore, the research team informed the participants and their parents (for participants <18) about the study’s aim, the voluntary nature of participation, and their freedom to refuse to participate at any time without consequence. Parental consent was obtained from all participants <18 years of age. The music students were given questionnaires and a sealable envelope (with a peel-off strip and stamp from the university) during their music lessons. Subsequently, they had the opportunity to leave the classroom to complete the questionnaire without the presence of a third individual (e.g., the teacher). The questionnaires were then placed in an envelope sealed by the students and given to the teacher who handed them over to the school principal. The school’s principal collected all envelopes and handed them to the research team.

### 4.3 Measures

The questionnaire contained items on motivational regulation, BPNS, parental involvement, and dropout tendency. All items were rated on a five-point Likert scale ranging from 1 (do not agree) to 5 (strongly agree).

#### 4.3.1 Motivational regulation

A shortened version of the self-regulation questionnaire (Ryan and Connell, 1989), adapted for instrumental music instruction, was used to determine motivational regulation. It captured intrinsic regulation (e.g., “I take part in instrumental music instruction because I enjoy it”; α = 0.80) and three forms of extrinsic motivation regulation styles: *Identified Regulation* (e.g., “I take part in instrumental music instruction because I want to perform with my friends”; α = 0.76); *Introjected Regulation*. Given that introjected regulation is associated with positive and negative aspects, this form of extrinsic motivation was divided into two subscales, *Positive Introjected Regulation* (e.g., “I take part in instrumental music instruction because I want to prove to myself that I am a good musician”; α = 0.83) and *Negative Introjected Regulation* (e.g., “I take part in instrumental music instruction because otherwise, I would have a guilty conscience”; α = 0.52); and *External Regulation* (e.g., “I take part in instrumental music instruction because I have to do it”; α = 0.71; see Table 1). Integrated regulation was not considered in this study, as it is highly correlated with intrinsic or identified regulation and can hardly be identified as a consistent factor on the continuum of self-determination (cf. Vallerand et al., 1992).

**Table 1:** Descriptive statistics.

	Music students (*N* = 140)			
	*M*	SD	*α*	Number of items
**Motivational regulation**				
Intrinsic	4.46	0.69	0.80	3
Identified	3.78	0.97	0.76	3
Positive introjected	2.74	1.03	0.83	5
Negative introjected	1.84	0.90	0.52	2
External	1.67	0.65	0.71	6
**Basic needs**				
Autonomy	3.76	1.04	0.71	2
Competence	4.65	0.47	0.58	3
Social Relatedness	4.65	0.51	0.75	3
Parental involvement	3.00	0.72	0.66	6
Tendency to dropout	1.22	0.66		1
Intention to change instrument	1.30	0.70		1
Intention to change teacher	1.02	0.15		1

Scale: 1 (do not agree) to 5 (strongly agree).

Based on SDT-related studies and the characteristics of motivation regulation styles, it is common to combine regulation styles into two scales: autonomous motivation and controlled motivation. In a study by Vansteenkiste et al. (2009), intrinsic motivation and identified regulation have been summarized as autonomous motivation, and extrinsic and introjected regulation as controlled motivation. Recent studies have shown that introjected regulation can be conceptually differentiated into positive (approach) and negative (avoidance) introjected regulation according to the degree of autonomy (Pelletier et al., 2013; Gagné et al., 2015); accordingly, positive introjected regulation was added to *autonomous motivation* and negative introjected regulation to *controlled regulation*. This resulted in the following formula: autonomous motivation = intrinsic + identified + positive introjected regulation, and controlled motivation = negative introjected + extrinsic regulation. Regarding construct validity, a confirmatory factor analysis (CFA) was performed using AMOS 26. Concerning autonomous motivation, the results of the CFA indicated an acceptable model fit, with the root mean square error of approximation (RMSEA) being slightly too high (χ^2^ = 86.461, df = 24, *p* < 0.001, CFI = 0.88, RMSEA = 0.12) and a good model fit for controlled motivation (χ^2^ = 2.948, df = 3, *p* < 0.40, CFI = 0.99, RMSEA = 0.05).

#### 4.3.2 Basic psychological needs satisfaction

To assess BPNS in instrumental music instruction, scales validated for the school sector by Thomas and Müller (2016) were used and adapted concerning content [autonomy (e.g., “My teacher lets me choose my own pieces of music”), competence (e.g., “If my teacher shows me something, I can do it much better afterwards”), and social relatedness (e.g., “I generally feel very comfortable in class”). Based on the use of short scales (Autonomy with two items) the reliability coefficients were satisfactory (α = 0.58–0.75). Given the intercorrelations among the three basic needs (see Table 1), the scales were combined with the overall BPNS scale. This practice was utilized in several other studies (e.g., Chen et al., 2015). Additionally, the CFA showed a good model fit for the single-factor solution for basic need satisfaction (χ^2^ = 12.925, df = 11, *p* < 0.29, CFI = 0.99, RMSEA = 0.04).

#### 4.3.3 Parental involvement

Zdzinski’s (1992) scale was used and items were adapted to assess the parental involvement in music (e.g., “My parents talk to me about music”; α = 0.66). CFA showed an acceptable model fit, with the RMSEA being only slightly too high (χ^2^ = 12.443, df = 7, *p* < 0.09, CFI = 0.95, RMSEA = 0.08).

#### 4.3.4 Tendency to dropout in music school

One item was used to assess the participants’ tendency to dropout (“If I could, I would like to stop attending music school immediately”), one to assess the participants’ intention to change instruments (“If I could, I would like to change music instrument immediately”), and one to assess participants’ intention to change their music teacher (“If I could, I would like to change my teacher immediately”).

## 5 Results

### 5.1 Descriptive statistics

Table 1 presents the descriptive statistics.

#### 5.1.1 Motivational regulation

The participants exhibited a very high degree of intrinsic motivation/regulation (*M* = 4.46; SD = 0.69) and a high degree of identified regulation (*M* = 3.78: SD = 0.97) to play/learn an instrument. Simultaneously, the participants exhibited low scores of negative introjected (*M* = 1.84; SD = 0.87) and external regulations (*M* = 1.67; SD = 0.65). Positive introjected regulation was rated as medium (*M* = 2.74; SD = 1.03). These results may indicate a ceiling effect.

#### 5.1.2 Basic needs

The participants scored high on social relatedness (*M* = 4.65; SD = 0.51) and competence (*M* = 4.65; SD = 0.47.) Support of Autonomy was also perceived as high (*M* = 3.76; SD = 1.04), but not at the level of the other needs.

#### 5.1.3 Parental involvement

Parental involvement was rated as medium (*M* = 3.00; SD = 0.72), and participants exhibited very low scores for tendency to dropout (*M* = 1.22; SD = 0.66), intention to change their instrument (*M* = 1.30; SD = 0.70), and intention to change teachers (*M* = 1.02; SD = 0.15).

### 5.2 Correlations

Table 2 provides an overview of the correlations between the most important variables. Tendency to dropout correlated the most with intrinsic motivation (*r* = −0.50, *p* < 0.01) and identified regulation (*r* = −0.33, *p* < 0.01). Additionally, a moderate correlation was found between tendency to dropout and parental involvement (*r* = −0.31, *p* < 0.01). Furthermore, parental involvement correlated with age (*r* = −0.25**). Therefore, older participants perceived less parental involvement. In addition, age was significantly correlated with support for autonomy (*r* = 0.39, *p* < 0.01). Therefore, older participants perceived more autonomy in their learning environments and had less of an intention to change instruments. Therefore, a correlation was found between age and intention to change the instrument (*r* = −0.22, *p* < 0.01).

**Table 2:** Spearman correlations among measured variables.

	1	2	3	4	5	6	7	8	9	10	11	12	13
1. Intrinsic motivation	–												
2. Identified regulation	0.46**	–											
3. Positive introjected regulation	0.13	0.42**	–										
4. Negative introjected regulation	−0.14	−0.01	0.36**	–									
5. External regulation	−0.24**	0.08	0.55**	0.48**	–								
6. Autonomy	0.19*	0.16	0.08	−0.04	−0.14	–							
7. Competence	0.36**	0.31**	0.05	0.15	−0.06	0.34**	–						
8. Social relatedness	0.40**	0.19*	0.03	−0.14	−0.13	0.28**	0.49**	–					
9. Parental involvement	0.21*	0.33**	**0.20**	−0.10	0.09	−0.01	0.09	0.03	–				
10. Tendency to dropout	−0.50**	−0.33**	−0.01	0.18*	0.09	0.10	−0.10	−0.24**	−0.31**	–			
11. Intention to change instrument	−0.26**	0.06	0.04	0.21*	0.18*	−0.05	−0.07	−0.19*	−0.22*	0.19*	–		
12. Intention to change teacher	−0.13	−0.02	0.01	−0.09	0.01	−0.09	−0.15	−0.26**	−0.06	−0.07	−0.06	–	
13. Age	0.13	0.03	0.06	0.06	−0.08	0.39**	0.06	0.13	−0.25**	−0.05	−0.22**	−0.01	–

*p < 0.05; **p < 0.01; significant correlations in bold.

### 5.3 Structural equation model

**Figure 2:**
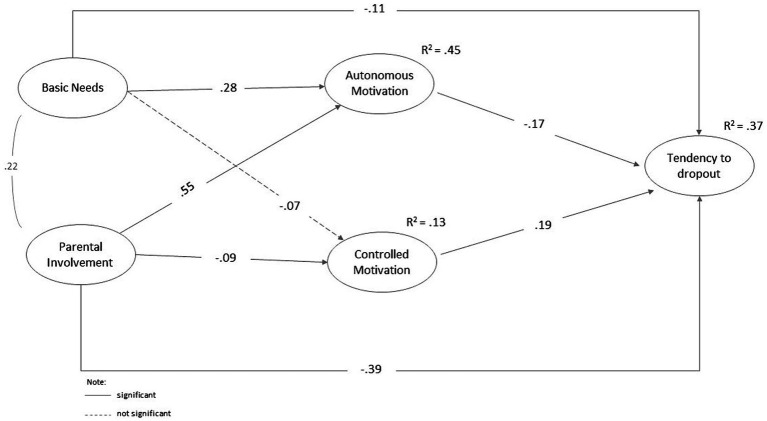
Structural equation model.

This study’s central aim was to predict music students’ dropout tendency of music school. Therefore, structural equation modeling (SEM) was conducted using AMOS 26 (Figure 2). The SEM showed an acceptable model fit, even if the CFI is somewhat low (χ^2^ = 664.969, df = 283, *p* < 0.01, CFI = 0.87, RMSEA = 0.09).

The best predictor for dropout tendency was parental involvement (β = −0.39, *p* < 0.01), followed by controlled motivation (β = 0.19, *p* < 0.01), autonomous motivation (β = −0.17, *p* < 0.01), and BPNS (β = −0.11, *p* < 0.05). Autonomous motivation (R^2^ = 0.45) was predicted by parental involvement (β = 0.55, *p* < 0.01) and BPNS (β = 0.28, *p* < 0.01). Controlled motivation (*R*^2^ = 0.13) was predicted by parental involvement (β = −0.09, *p* < 0.05). Furthermore, the BPNS does not provide a significant explanatory account of controlled motivation (β = −0.07, *p* = 0.11). Thus, the BPNS and parental involvement can explain the tendency to drop out, mediated by the two motivational styles of autonomous and controlled motivation. In addition to this mediation effect, parental involvement can also directly and substantially explain the dropout tendency (β = −0.39, *p* < 0.01).

## 6 Summary and discussion

This study aimed to explain the tendency to drop out of music school through the BPNS in lessons, parental involvement in music, and the quality and quantity of motivation when playing instruments. This contributes to reducing the research gap on motivation in extracurricular (instrumental) music settings. Particularly, the relevance of parental involvement has not yet been examined in previous studies (Evans et al., 2013).

This study showed that the participants were significantly more autonomously motivated to learn and play instruments and were slightly regulated in a controlled manner. The high level of autonomous motivation was a contributing factor and the voluntary nature of music school lessons in Austria also played a role. Furthermore, basic psychological needs were largely satisfied during the music lessons. Students rated parental involvement on a medium level (*M* = 3.00; SD = 0.72) and the tendency to dropout and the tendency to change musical instruments or teachers were rather low.

BPNS correlated with intrinsic and identified motivation (*r* = 0.19, *p* < 0.05 to *r* = 0.40, *p* < 0.01), confirming the *first hypothesis*. In the present study, competence and social relatedness were significantly more correlated with autonomous forms of motivation than autonomy. As expected, the BPNS provided little explanatory contribution to the controlled forms of motivation. Social relatedness was negatively associated with controlled forms of motivation. These findings are consistent with those of other studies examining the importance of the BPNS in the prediction of motivational quality (e.g., Vandenkerckhove et al., 2019; Kaiser et al., 2020; Müller et al., 2021; Ryan, 2023). However, the association between needs and autonomous forms of motivation may vary depending on the setting.

The results also confirmed the *second hypothesis*. Moderate correlations were found between parental involvement and the autonomous forms of motivation, intrinsic (*r* = 0.21, *p* < 0.05) and identified regulation (*r* = 0.33, *p* < 0.01). This is consistent with McPherson and O’Neill’s (2010) findings. Accordingly, parental involvement played a central role in explaining intrinsic and identified motivation (cf. Leung and McPherson, 2010; McPherson and Hendricks, 2010; McPherson and O’Neill, 2010).

Further, the *third hypothesis* was confirmed. Autonomous forms of motivation were negatively correlated and controlled motivation was positively correlated with the tendency to drop out. Intrinsic (*r* = −0.50) and identified regulations (*r* = −0.33, *p* < 0.01) were particularly important predictors. This is consistent with the theory that negative introjected regulation (avoidance) correlates significantly and positively with the tendency to dropout (*r* = 0.17*). This means that those who act to avoid negative emotions, such as a guilty conscience or feelings of guilt, are more likely to consider dropping out of music schools.

No separate hypothesis was formulated concerning age. However, more older participants rated the BPNS in lessons more favorably than younger participants. Among other factors, this could be attributed to self-selection processes; young individuals or adult music students who are dissatisfied with their learning environment may have already departed from music schools because of lower satisfaction of their needs or because of the shift in interests. Parental involvement is no longer relevant for older students (*r* = −25; *p* < 0.01), as they seem to attend music school or play instruments largely independent of parental behavior.

The *fourth hypothesis* concerned the direct and indirect relationships among BPNS, parental involvement, autonomous and controlled motivation, and the tendency to drop out of music school. We used a structural equation model to test this. Corresponding with expectations, controlled motivation tended to lead to dropping out, whereas autonomous motivation positively affected remaining at a music school (cf. StGeorge, 2004). The BPNS explained the tendency to drop out as being mediated by autonomous motivation. The direct path of BPNS and tendency to dropout is comparatively low (ß = −0.11), whereby the negative sign was to be expected from the trend. This finding was similar to those in other studies on dropout in educational settings (e.g., Vallerand et al., 1997). Parental involvement explains the tendency to dropout via autonomous motivation but also explains dropout tendency directly. This means that music students who are supported and receive attention from their parents regarding learning an instrument are autonomously motivated and tend to continue attending music school. This finding is consistent with those of previous studies (Leung and McPherson, 2010; McPherson and Hendricks, 2010; McPherson and O’Neill, 2010) and confirms the importance of the parental home for self-determined motivation and resistance to learning a musical instrument. In addition, McPherson and colleagues` interviews with parents (2012) showed that family dynamics (e.g., practice supervision, beliefs and values about music and family’s music experiences) had an impact on children’s musical development.

The structural equation model showed that the predictors BPNS and parental involvement in particular can explain the tendency to drop out of music lessons, also mediated by autonomous and controlled motivations.

### 6.1 Limitations and further research

This study had several limitations, including the sample size, research design, and scales used. Regarding sample composition, this study utilized a more or less self-selected sample, and only three music schools in a region were included. Owing to the estimated sample size using G*Power software and the present sample size, these limitations should not have a substantial effect. Furthermore, the possibility of sampling bias cannot be excluded, with a greater proportion of students who were intrinsically motivated participating in the survey. However, precise information on the response rate is not available. Overall, conclusions should be drawn carefully as the current study did not employ a longitudinal research design. Future studies should provide additional insights into the development of students’ tendencies to drop out and their motivation to remain in music schools. A longitudinal study would allow for determining actual dropouts and not just the estimated tendency for such a behavior. Therefore, further studies are required to determine why music students quit (e.g., through interviews and a qualitative approach). Regarding the assessment of parental involvement, only music students’ perceptions of parental involvement were examined in this study, without questioning the parents. Parents were not questioned concerning their involvement and the general “music culture” in the families, which has been addressed by McPherson et al. (2012) and might be included in future studies. Additionally, it would be advantageous to include teachers’ perceptions in future studies.

The quality of the measurements can also be cited as a limitation in some cases. Negative introjected regulation was measured by using only two items and the tendency to dropout was assessed with one item. Consequently, the reliability of these measures is low, which somewhat reduces the significance of the findings.

This study highlighted future research interests. First, an examination of the satisfaction of basic needs revealed that autonomy was perceived to be lower than competence and social relatedness. One potential explanation for this could be the examination of the correlation between the students’ preferred musical genre and the genre that dominated during music instruction. Classical genres and folk music commonly predominate in Austrian music schools. Therefore, an insufficient fit could harm autonomy and consequently lead to dropping out (see also Feshchanka, 2021). Second, music schools in this region may not be comparable to instrumental music education offered through private instrument tuition. Private music instruction partially employs more popular musical genres for their students, and some may even call themselves a “School of Rock.” These schools do not specialize in classical or folk music. The fit between the preferred genre and genre played in music schools may be better for private music instruction. Third, the question arises as to the extent to which formal settings influence motivation and dropout. Music theory is mandatory in Austrian music schools (particularly when taking instrumental exams). Based on these considerations, undesired (performance) pressure could arise, which might negatively affect the motivation to play an instrument and attend music school.

## Data availability statement

The data can be obtained from the authors on request.

## Ethics statement

The studies involving humans were approved by Institutional Review Board of the University of Klagenfurt. The studies were conducted in accordance with the local legislation and institutional requirements. Written informed consent for participation in this study was provided by the participants’ legal guardians/next of kin.

## Author contributions

MW: Writing – original draft, Writing – review & editing. VN-G: Writing – original draft, Writing – review & editing. FM: Writing – original draft, Writing – review & editing.
